# SLAMF8 can predict prognosis of pan-cancer and the immunotherapy response effectivity of gastric cancer

**DOI:** 10.18632/aging.205850

**Published:** 2024-05-22

**Authors:** Guangyao Li, Qijiao Li, Miaomiao Ping, Ziying Jiao, Xingxing Wang, Juan Cheng, Jizheng Guo, Ya Cheng

**Affiliations:** 1 Department of Gastrointestinal Surgery, The Second People’s Hospital of Wuhu, Wuhu 241000, Anhui, China; 2 School of Basic Medical Sciences, Anhui Medical University, Hefei 230032, Anhui, China; 3 Department of Emergency Surgery, The First Affiliated Hospital of Anhui Medical University, Hefei 230032, Anhui, China

**Keywords:** SLAMF8, pan-cancer, biomarker, TME, immunotherapy

## Abstract

SLAMF8, the eighth member of the Signaling Lymphocytic Activation Molecule Family (SLAMF), functions in the regulation of the development and activity of diverse immune cells as a costimulatory receptor within the SLAMF family. Studies had revealed that SLAMF8 is expressed higher in several autoimmune inflammation diseases and tumors. Nevertheless, the connection between SLAMF8 and pan-cancer remains undisclosed. The research investigated the correlation between SLAMF8 and various factors including the immune microenvironment, microsatellite instability, immune novel antigen, gene mutation, immune regulatory factors, immune blockade TMB, and immune or molecular subtypes of SLAMF8 in verse cancer types. Immunohistochemistry was ultimately employed to validate the presence of the SLAMF8 gene in various tumor types including hepatocellular carcinoma, prostate adenocarcinoma, and kidney renal clear cell carcinoma. Furthermore, the relationship between SLAMF8 expression and the therapeutic efficacy of the PD1 blockade agent, Sintilimab, treatment in gastric cancer was validated. The result of differential analysis suggested that SLAMF8 was over-expressed in pan-cancer compared with paracancerous tissues. The analysis of survival indicated a connection between SLAMF8 and the overall prognosis in different types of cancers, where higher levels of SLAMF8 were found to be significantly linked to unfavorable outcomes in patients but favorable outcome of immunotherapy in gastric cancer. Significant correlations were observed between SLAMF8 levels and pan-cancer tumorigenesis, tumor metabolism, and immunity. As a result, SLAMF8 may become an important prognostic biomarker in the majority of tumors and a hopeful gene target for immunotherapy against gastric cancer.

## INTRODUCTION

The International Agency for Research on Cancer (IARC) disclosed that around 19.3 million new cancer cases were documented worldwide in 2020, and despite ongoing advancements in cancer treatment strategies, the outlook for cancer prognosis remained pessimistic [1, 2]. Enhancing cancer management relies on the improvement of cancer diagnosis and prognosis, making it imperative to explore new biomarkers for these purposes.

The eighth member of the Signaling Lymphocytic Activation Molecule Family (SLAMF), known as SLAMF8 (CD353), is a costimulatory receptor within the SLAMF family. It plays a regulatory role in the development and function of diverse immune cells, including T lymphocytes, B cells, neutrophils, dendritic cells, macrophages, and eosinophils [3, 4]. Recent studies had revealed that SLAMF8 is more highly expressed in several autoimmune inflammation diseases and tumors, such as inflammatory bowel disease, human post-renal transplantation, glioma and anaplastic large cell lymphoma [5–8], which suggested that SLAMF8 plays a key role in immune-related inflammation response and the tumorigenesis and tumor progress of some cancers. However, up to date, there are no researches that confirmed the expression pattern, prognostic significance, and biological role of SLAMF8 across various cancers. In recent years, bioinformatics analysis has played a crucial role in the molecular classification and identification of biomarkers in cancers [9, 10]. In this study, a thorough analysis of SLAMF8 was conducted to investigate its abnormal expression, its relationship with pan-cancer prognosis and the immune microenvironment, and to verify its ability to predict the effectiveness of immunotherapy in gastric.

## MATERIALS AND METHODS

### Data sources

The gene expression matrix and clinical information from both tumor and non-tumorous samples were sourced from the TCGA data resource, GTEx data resource, and UCSC data resource. Expression data for tumor cell lines were extracted from the CCLE data resource. Moreover, the TIMER data resource (https://cistrome.shinyapps.io/timer/) was utilized to extract data on immune invasion cell scores in pan-cancer.

### SLAMF8 expression in pan-cancer

R software was utilized to analyze variations in SLAMF8 expression levels between tumor tissues and normal tissues. Subsequently, the distinctions in SLAMF8 expression levels across different normal tissues and various tumor cell lines were examined using the Kruskal-Wallis test. Violin plots depicting the data were generated using the ggplot R package.

### Immunohistochemistry of paraffin sections

48 pairs of STAD and paracancerous tissue specimens were obtained from Wuhu Second Hospital’s specimen bank, which were recurrent after radical gastric cancer surgery from September 2022 to October 2023. These patients belonged to stage II and III and received 6 cycles of conventional XELOX or SOX chemotherapy followed by paclitaxel combined with Sintilimab treatment in the Second Hospital of Wuhu City after recurrence. The research received approval from Wuhu Second Hospital’s Ethics Committee, and all patients gave informed consent (2023-KY-010). Besides, 30 pairs of HCC, PRAD and KRIC cancer tissues and paracancerous tissue specimens were obtained in the similar way. Anti-SLAMF8 rabbit polyclonal antibody was purchased from Abmart company (PHN8370) and diluted it at a 1:100 ratio, following the same specific immunohistochemistry methods and the way of assigning scores as described in a previously published article [11, 12]. ImageJ software was utilized for scoring, followed by statistical analysis and visualization based on the obtained scores.

### SLAMF8 expression and prognosis of pan-cancer

The method of univariate survival analysis was employed to examine the correlation between SLAMF8 expression and patient survival. The Kaplan-Meier model was used to assess survival in pan-cancer across varying levels of SLAMF8 expression. To categorize the levels of SLAMF8 expression in tumors and nearby noncancerous tissues, a bipartite approach was employed to establish groups with high and low expression. Univariate Cox survival analysis was conducted using R package survival (version 3.2-7).

### SLAMF8 expression with immune microenvironment in pan-cancer

In cancer, the presence of tumor-infiltrating lymphocytes is a reliable indicator of both sentinel lymph node status and survival. The prediction of cancer outcomes is linked to immune scores and stromal scores. Following the approach used in a prior investigation [13], we examined the association between gene expression and immune cell scores using the R package ESTIMATE (version 1.0.13). A correlation was considered significant if the *p*-value was less than 0.05 and the R-value was greater than 0.20, indicating a positive connection.

### SLAMF8 expression with immune neoantigens and immune checkpoints genes

The mutation of genes within malignant cells, such as point mutations, gene fusions, and deletion mutations, can encode neoantigens. The neoantigens’ docking affinity score was computed according to antigenic epitopes comprising 8 to 11 amino acids and epitopes with a score below 500 nm were categorized as neoantigens. Neoantigens were sorted based on their docking affinity, variant allele frequency, and antigenicity index values. Consistent with the research methodology of a previous study [13], the count of neoantigens in tumor samples were measured with ScanNeo tool. Additionally, the correlation between the expression of SLAMF8 and the quantity of antigens was examined. Furthermore, an investigation was conducted to examine the correlation between the expression of SLAMF8 and the top 40 immune checkpoint genes. The expression levels of immune checkpoint genes were individually abstracted, and their correlation with SLAMF8 expression was determined. Remarkably positive correlations were indicated by *p* < 0.05 and R > 0.20.

### SLAMF8 expression with tumor mutational burden and microsatellite instability

Tumor mutational burden (TMB) reflects the quantity of mutations within a tumor cell. The TMB for each tumor sample was independently calculated using Spearman’s rank correlation coefficient. Microsatellite instability (MSI) involves the emergence of new microsatellite alleles in comparison to normal tissue, where any change in the length of a microsatellite is caused by the insertion or deletion of a repeat unit. The correlation between SLAMF8 expression and MSI was assessed through Spearman’s rank correlation coefficient.

### SLAMF8 expression with mismatch repair genes and DNA methyltransferases

Mismatch repair is an intracellular mechanism responsible for correcting mismatch errors during DNA replication. Loss of function in key genes of this mechanism leads to unrepaired DNA replication errors, resulting in higher levels of somatic mutations. The association between SLAMF8 expression and five mismatch repair genes (MLH1, MSH2, MSH6, PMS2, EPCAM) was examined using expression profile data from TCGA. DNA methylation, a chemical modification of DNA capable of influencing epigenetic inheritance and controlling gene expression without altering the DNA sequence, was analyzed in this study. Visualization analysis was conducted to explore the relationship between SLAMF8 expression and the expression of four methyltransferases using ggplot. Correlations were considered significant and positive when *p* < 0.05 and R > 0.20.

### Gene set enrichment analysis of SLAMF8 in pan-cancer

Gene set enrichment analysis (GSEA) is an analytical approach that compares genes with predefined gene sets to investigate their expression status within a specific functional gene set. The evaluation determines if the expression status is significantly related to the biological process, molecular function, or cellular component [14]. Kyoto Encyclopedia of Genes and Genomes (KEGG) is a comprehensive database that integrates genomic, chemical, and systematic functional information. For GSEA analysis, another dataset used is the molecular signatures database (MsigDB) [15], with the Hallmark gene set employed in this analysis. Pathways were considered significantly enriched if they met the sub-conditions of |NES| >1, *p*-value < 0.05, and FDR < 0.25 as the GSEA threshold.

### TISIDB analysis

The TISIDB website (http://cis.hku.hk/TISIDB/index.php), comprising 998 immune-related anti-tumor genes extracted from 4176 records in 2530 publications, provides a platform for scholars to analyze target genes in the tumor–immune interplay using high-throughput data analysis or literature mining [16]. In our study, TISIDB was utilized to generate heat maps, allowing us to explore the Spearman relationship between SLAMF8 expression levels and immunomodulators as well as immune cells in various cancer types. Additionally, we examined the association between SLAMF8 expression and immune subtypes or molecular subtypes across human cancers using TISIDB, considering a *p*-value < 0.05 as statistically significant.

### Single cell analysis

The Tumor Immune Single Cell Hub 2 (TISCH2, http://tisch.comp-genomics.org/) is a single-cell RNA sequencing (scRNA-seq) database that specifically focuses on the tumor microenvironment (TME). This database encompasses 79 datasets and provides single-cell transcriptome profiles for more than 2 million cells. Additionally, the IMMUcan scDB (https://immucanscdb.vital-it.ch/) comprises 144 datasets covering 56 different cancer types, annotated in 50 fields with precise clinical, technological, and biological information. Another online database, TIGER (http://tiger.canceromics.org/#/), contains bulk transcriptome gene expression data for 1508 tumor samples across 8 cancer types, including clinical immunotherapy information from 20 published studies, as well as 11,057 tumor/normal samples from 33 cancer types. In our study, we systematically utilized TISCH2, the IMMUcan database (search by gene and annotation major), and TIGER to investigate TME heterogeneity.

## RESULTS

### SLAMF8 is aberrantly expressed in various cancers

**Figure 1:**
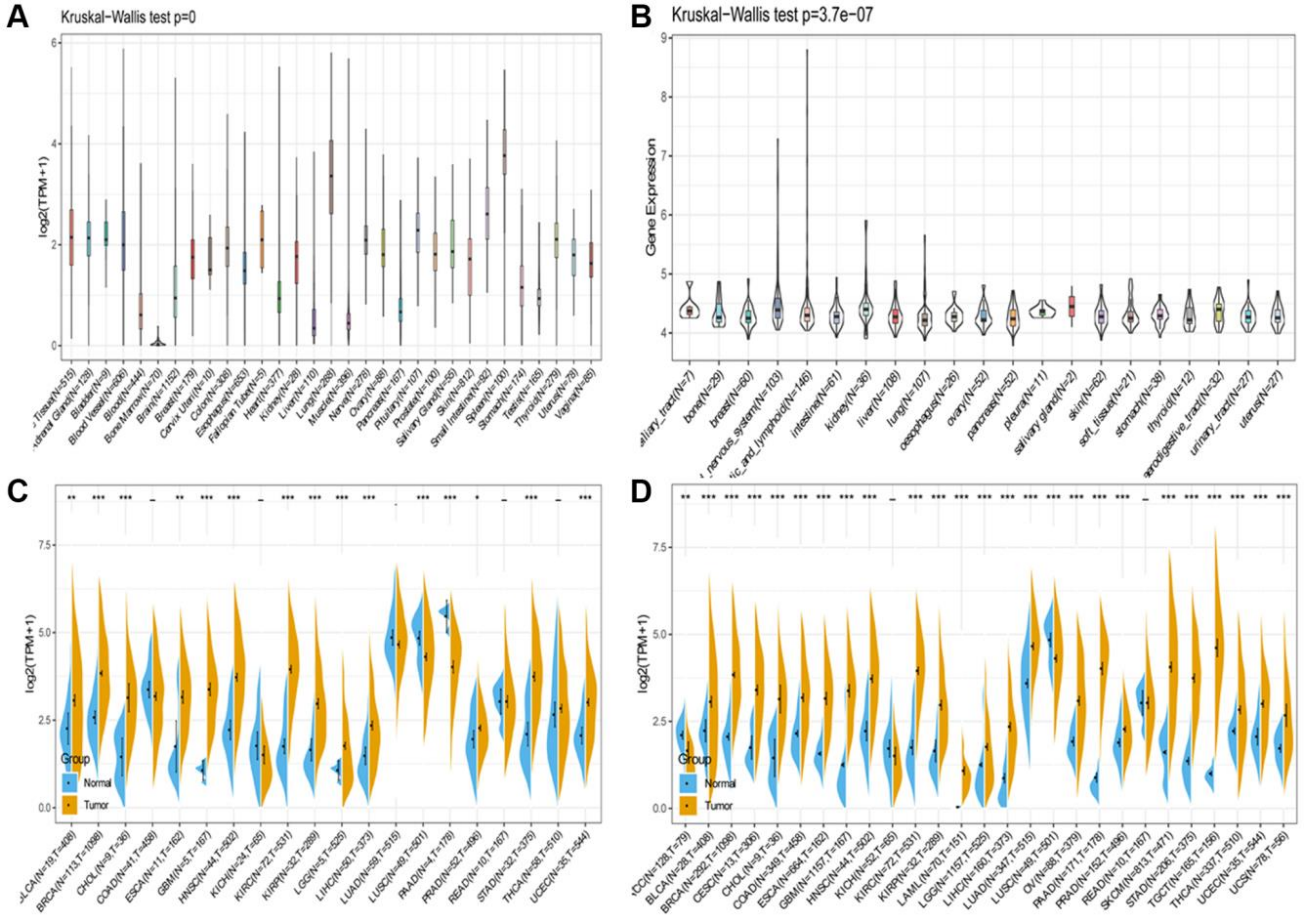
**SLAMF8 expression in pan-cancer.** (**A**) Expression levels of SLAMF8 in a database contain 31 normal tissues, obtained from the GTEx. (**B**) Expression levels of SLAMF8 in a dataset containing 21 tissues in tumor cell lines, collected from the CCLE. (**C**) Expression levels of SLAMF8 in tumor and paired adjacent noncancerous tissues containing 20 tissues from TCGA, ^*^P < 0.05, ^**^P < 0.01, and ^***^P < 0.001. (**D**) SLAMF8 expression difference in 27 tumors integrating data of normal tissues in GTEx database and data of TCGA tumor tissues, ^*^P < 0.05, ^**^P < 0.01, and ^***^P < 0.001.

AS the Figure 1A shown, GTEx data resources were used to describe the trend of SLAMF8 expression in 31 types normal tissues. The SLAMF8 expression data of 21 types tumor cell lines retrieved from CCLE data resources were analyzed and the result was shown in Figure 1B. The expression levels of SLAMF8 in TCGA were compared between cancer tissues and adjacent tissues. The results, displayed in the form of a box diagram in Figure 1C, demonstrated that the expression of SLAMF8 was higher in the majority of cancer types. Furthermore, we integrated the mRNA content data of SLAMF8 for nontumorous tissues from GTEx data resource as well as normal and malignant tissues from TCGA dataset to compare the difference in SLAMF8 expression in 27 types tumors (Figure 1D), and results confirmed that SLAMF8 expression were upregulated in the great mass of tumor types.

### SLAMF8 is related to prognosis in various cancers

**Figure 2:**
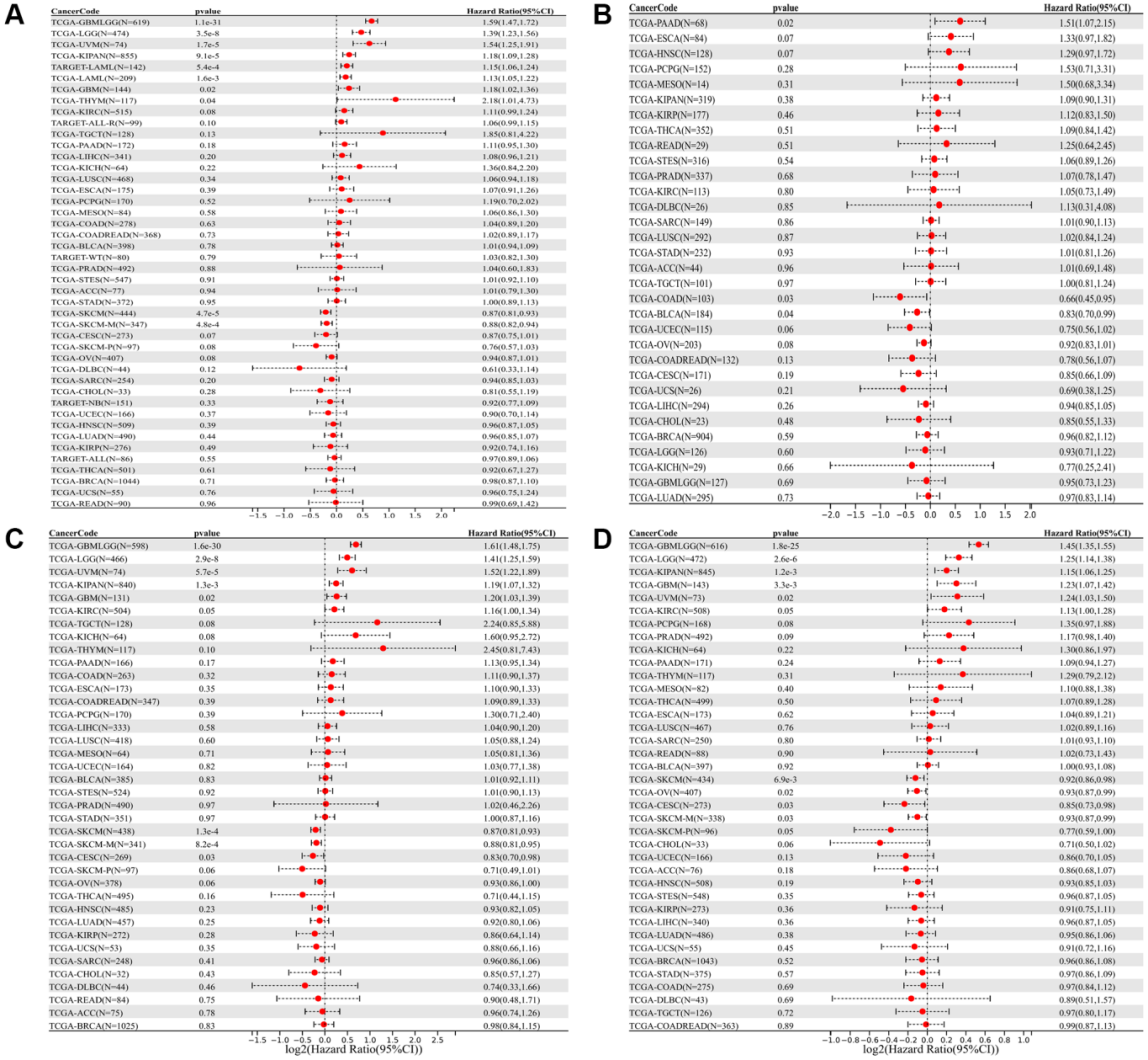
Forest plot of the relationship between SLAMF8 expression and OS (**A**), DFI (**B**), DSS (**C**), and PFI (**D**) time in days, utilizing univariate survival analysis, across 33 types of tumors.

We investigated the correlation of SLAMF8 mRNA levels with overall survival (OS), disease-specific survival (DSS), disease-free survival (DFS), and progression-free interval (PFI) across 44 cancer types. The prognostic value of SLAMF8 was evaluated using Kaplan-Meier survival curves and Cox proportional hazards models. Forest plots were used to present the results of Cox regression analyses and the association of SLAMF8 with OS, DFI, DSS, and PFI. In GBMLGG, LGG, UVM, KIPAN, LAML, GBM, and THYM, we found that SLAMF8 expression is positively correlated with overall survival (OS), whereas it is negatively linked to OS in SKCM and SKCM-M (Figure 2A). Additionally, SLAMF8 expression was negatively correlated with DFI in COAD and BLCA, and positive in PAAD (Figure 2B). Furthermore, SLAMF8 expression showed a positive correlation with DSS in GBMLGG, LGG, UVM, KIPAN, GBM, and KIRC, and negative in SKCM, SKCM-M, and CESC (Figure 2C). Lastly, SLAMF8 expression was negatively correlated with PFI in SKCM, OV, CESC, and SKCM-P, and positive in GBMLGG, LGG, KIPAN, GBM, UVM, and KIRC (Figure 2D).

**Figure 3:**
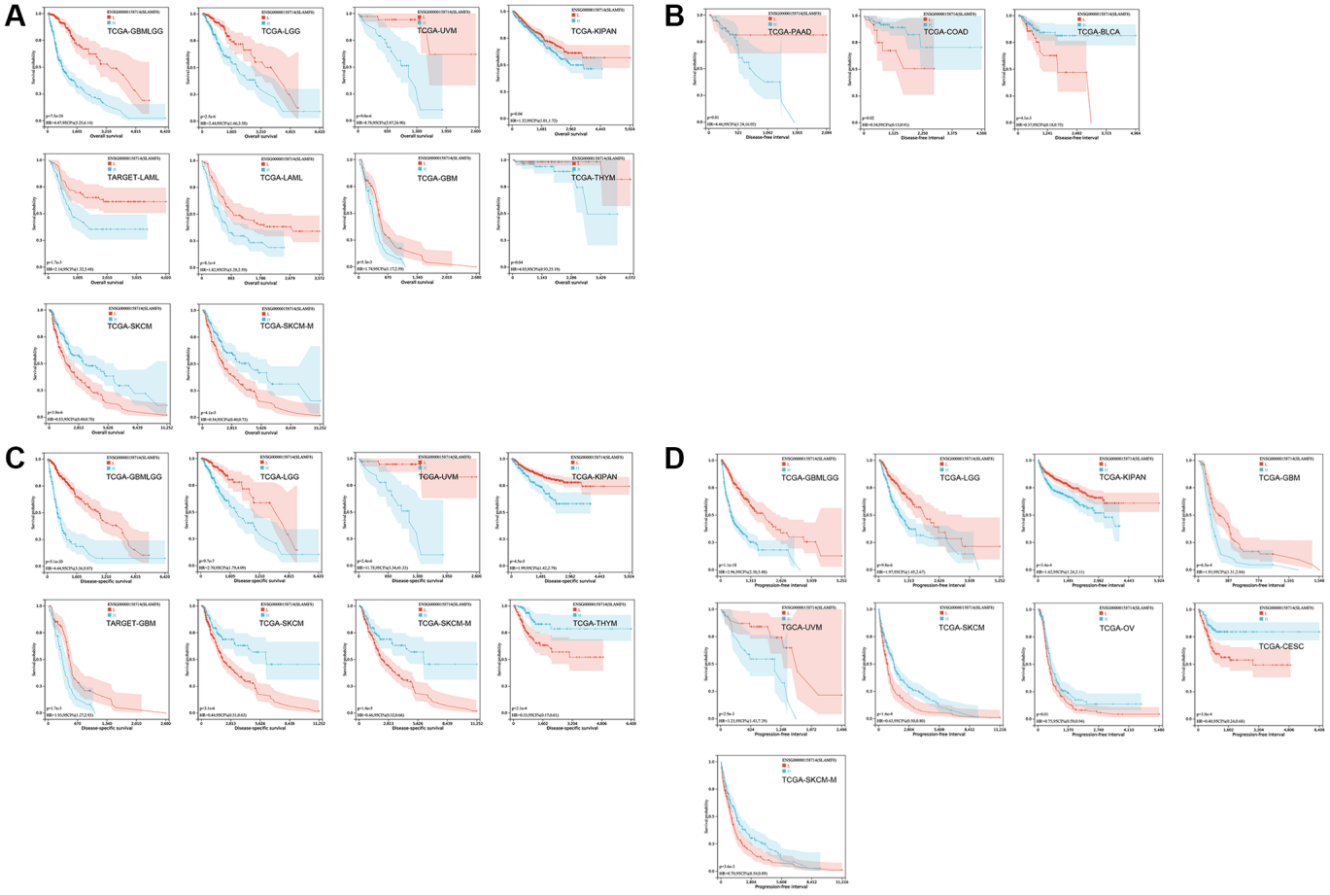
**The survival curve of SLAMF8 in pan-cancer using Kaplan-Meier analysis and log-rank test.*** P*-values < 0.05 were considered and shown. (**A**) The survival curve of SLAMF8 for OS in 10 kinds of tumor. (**B**) The survival curve of SLAMF8 for DFI in 3 kinds of tumor. (**C**) The survival curve of SLAMF8 for DSS in 8 kinds of tumor. (**D**) The survival curve of SLAMF8 for PFI in 9 kinds of tumor.

The Kaplan-Meier (KM) curve results revealed that elevated SLAMF8 expression was associated with poorer overall survival (OS) in GBMLGG, LGG, UVM, KIPAN, LAML, GBM and THYM, while indicating improved OS in SKCM and SKCM-M (Figure 3A). Moreover, increased SLAMF8 mRNA levels were linked to inferior DFI in COAD and BLCA, and worse in PAAD (Figure 3B). Heightened SLAMF8 expression was associated with enhanced DSS in SKCM, SKCM-M and THYM, and worse in GBMLGG, LGG, UVM, KIPAN and GBM (Figure 3C). Lastly, over-expressed SLAMF8 suggested improved PFI in SKCM, OV, CESC and SKCM-M, and worse in GBMLGG, LGG, KIPAN, GBM and UVM (Figure 3D).

### SLAMF8 is related to the infiltration of immune cells and the surrounding environment in various cancers

**Figure 4:**
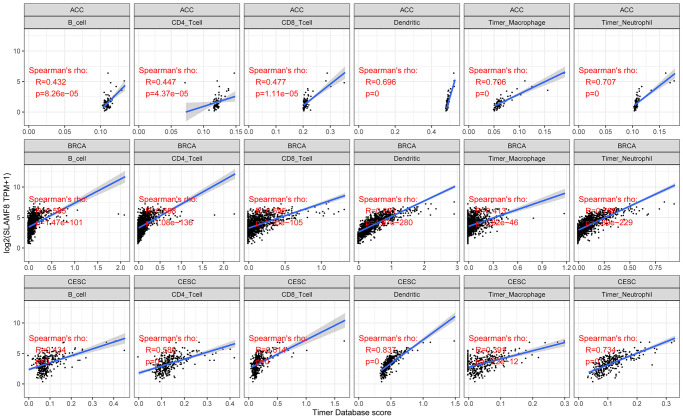
Analysis for correlation between SLAMF8 expression and tumor immune infiltration in top 3 cancers (ACC, BRCA, and CESC).

**Figure 5:**
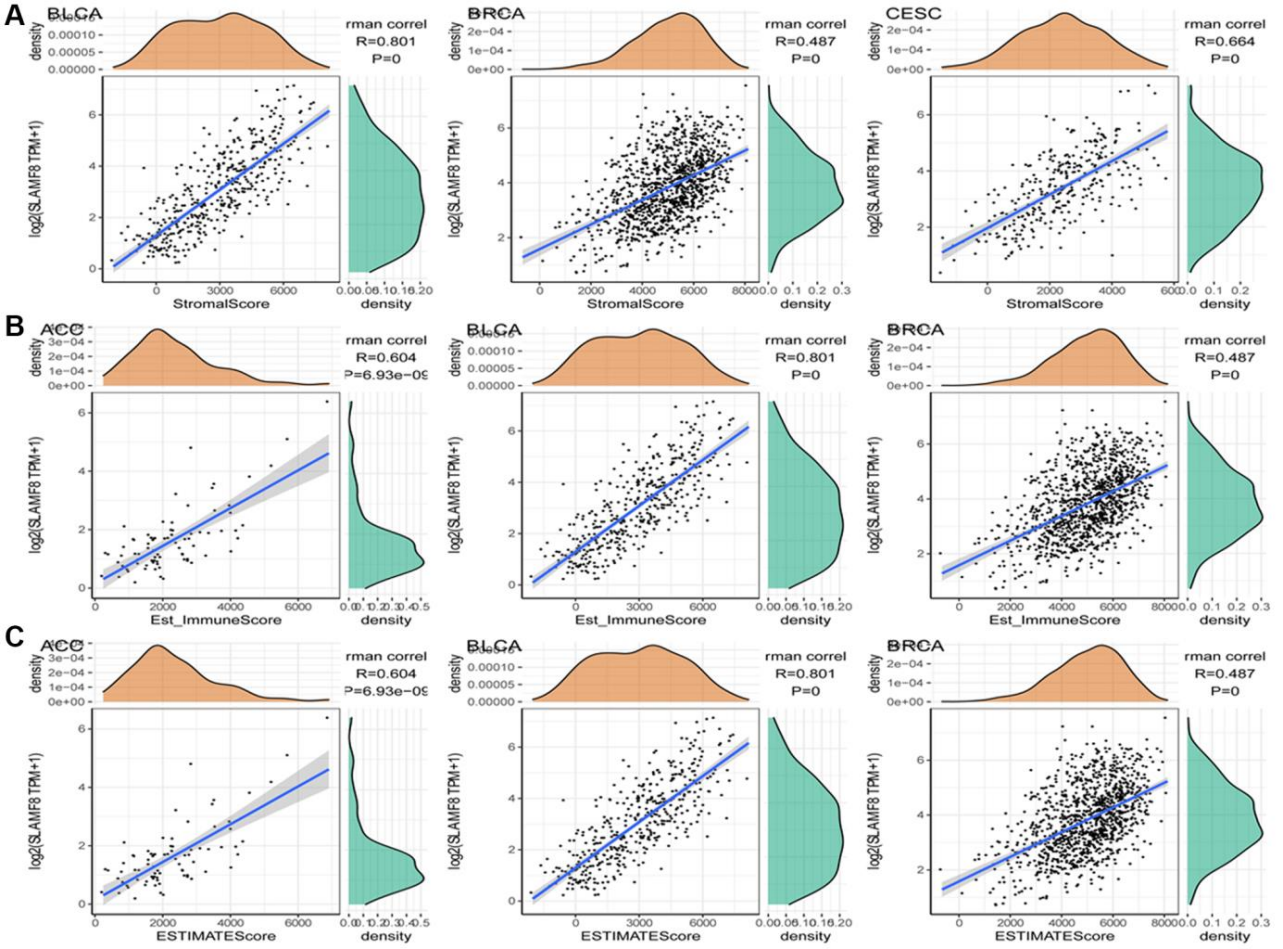
**Analysis for correlation between SLAMF8 expression in pan-cancer and tumor microenvironment.** (**A**) Correlation analysis between SLAMF8 expression and stromal score in top 3 cancers. (**B**) Correlation analysis between SLAMF8 expression and immune score in top 3 cancers. (**C**) Correlation analysis between SLAMF8 expression and estimate immune score in top 3 cancers.

In cancers, tumor-infiltrating lymphocytes serve as autonomous prognostic indicators. The data from our study revealed a notable correlation between SLAMF8 and the extent of immune infiltration in various cancers, specifically ACC, BRCA, and CESC (as shown in Figure 4). SLAMF8 expression level exhibited a significant correlation with all six categories of tumor-infiltrating lymphocytes, encompassing B cells (R = 0.432, 0.585, and 0.434, *P* < 0.001), CD4+ T cells (R = 0.447, 0.658, and 0.589, *P* < 0.001), CD8+ T cells (R = 0.477, 0.595, and 0.614, *P* < 0.001), dendritic cells (R = 0.696, 0.831, and 0.837, *P* < 0.001), macrophages (R = 0.706, 0.413, and 0.391, *P* < 0.001), and neutrophils (R = 0.707, 0.785, and 0.734, *P* < 0.001) in ACC, BRCA, and CESC. Moreover, the stromal and immune scores of cancer samples were examined using the R package ESTIMATE to explore the influence of SLAMF8 on the progression of tumors in the tumor immune microenvironment. Among the 33 types of cancer, the findings indicated that BLCA (R = 0.801, *P* < 0.001), BRCA (R = 0.487, *P* < 0.001), and CESC (R = 0.664, *P* < 0.001) had the strongest correlation between SLAMF8 and the stromal score. This was shown in Figure 5A. Among the 33 tumors, the three most prominent cancer types showing a strong connection between SLAMF8 and the immune score were ACC (R = 0.604, *P* < 0.001), BLCA (R = 0.801, *P* < 0.001), and BRCA (R = 0.487, *P* < 0.001) (Figure 5B). Additionally, ACC (R = 0.604, *P* < 0.001), BLCA (R = 0.801, *P* < 0.001), and BRCA (R = 0.801, *P* < 0.001) exhibited the highest correlation between SLAMF8 and the ESTIMATE score (Figure 5C), making them the top cancer types.

**Figure 6:**
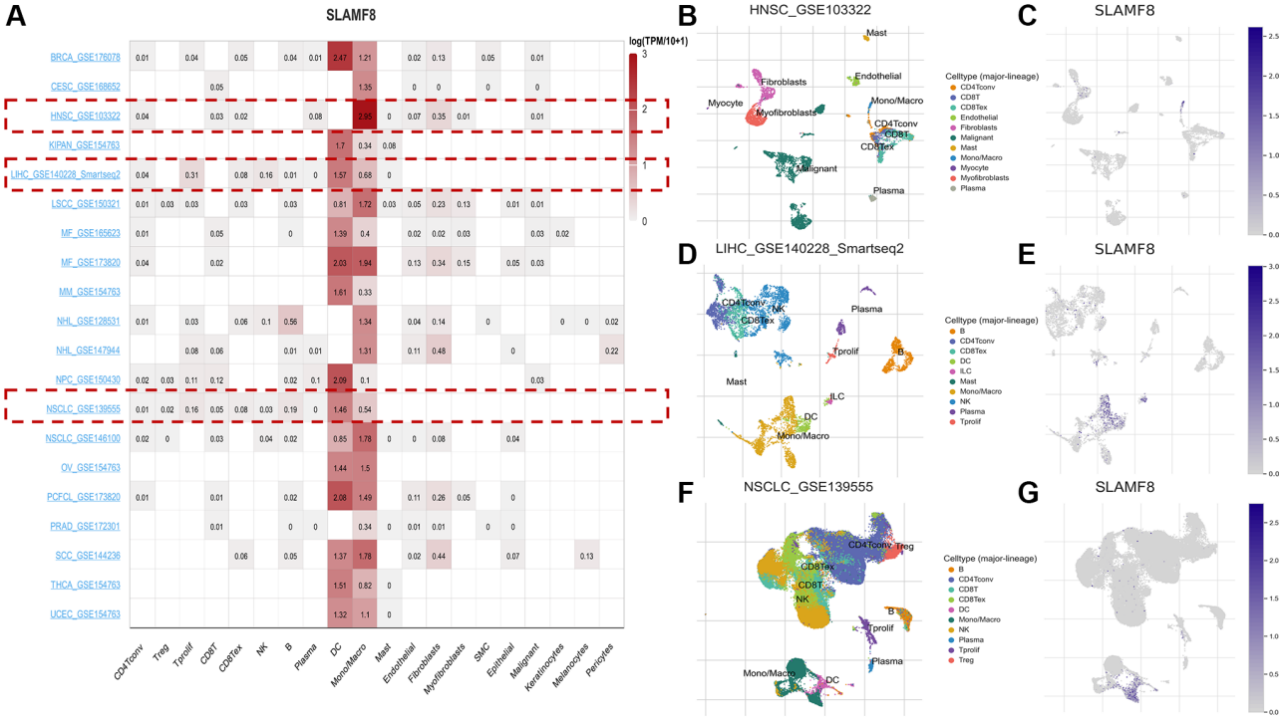
**The expression of SLAMF8 in TME-related cells based on TISCH2 database.** (**A**) Correlation between the SLAMF8 expression and the TME in TISCH database. (**B**, **C**) The cell types and their distribution in the HNSC_GSE1033227 dataset. (**D**, **E**) The cell types and their distribution in the LIHC_GSE140228_Smartseq2 dataset. (**F**, **G**) The cell types and their distribution in the NSCLC_GSE139555 dataset.

**Figure 7:**
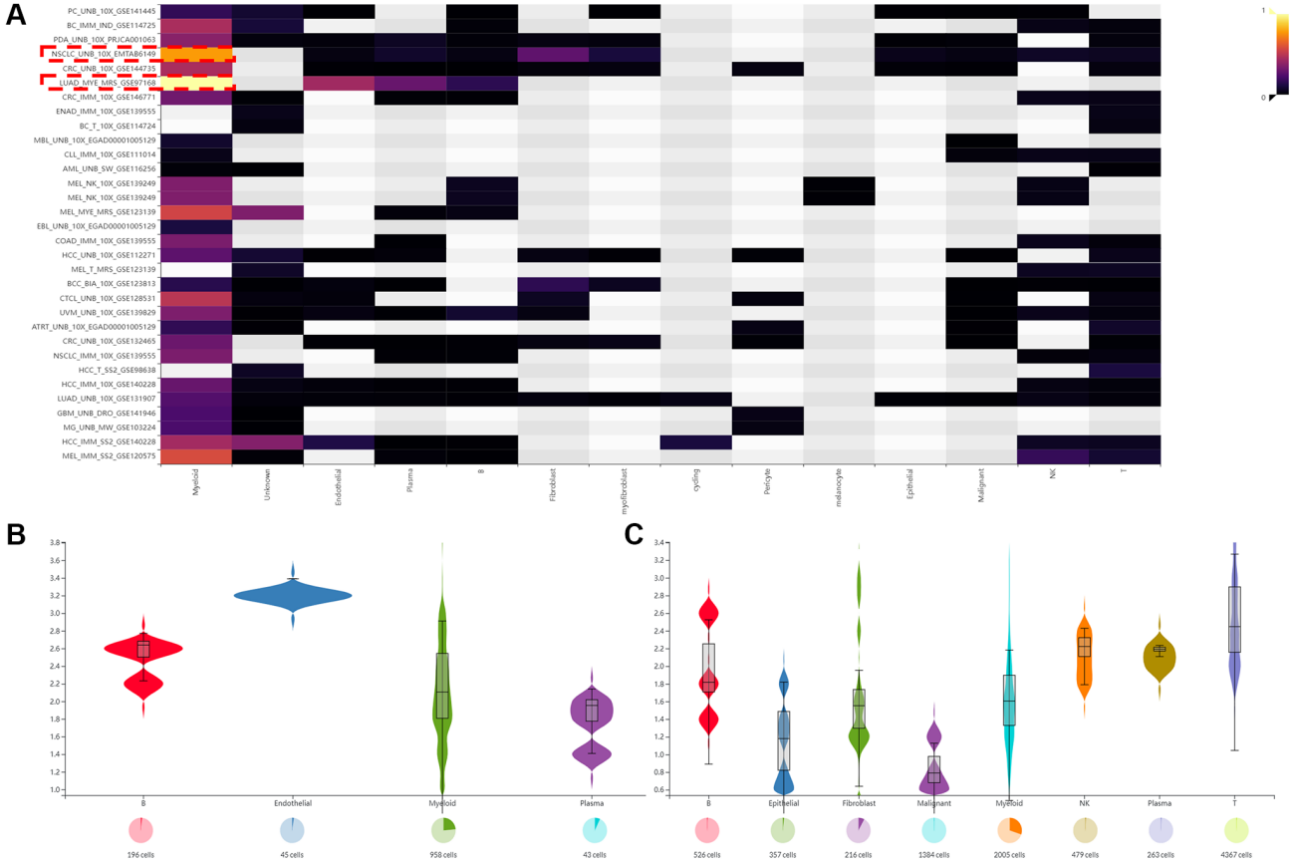
**The expression of SLAMF8 in TME based on IMMUcan SingleCell RNAseq database.** (**A**) The distribution of SLAMF8 expression in various datasets downloaded from IMMUcan SingleCell RNAseq database. (**B**, **C**) The expression of SLAMF8 in various TME cells in NSCLC_UNB_10x_EMTAB6149 and LUAD_MYE_MRS_GSE97168 datasets.

**Figure 8:**
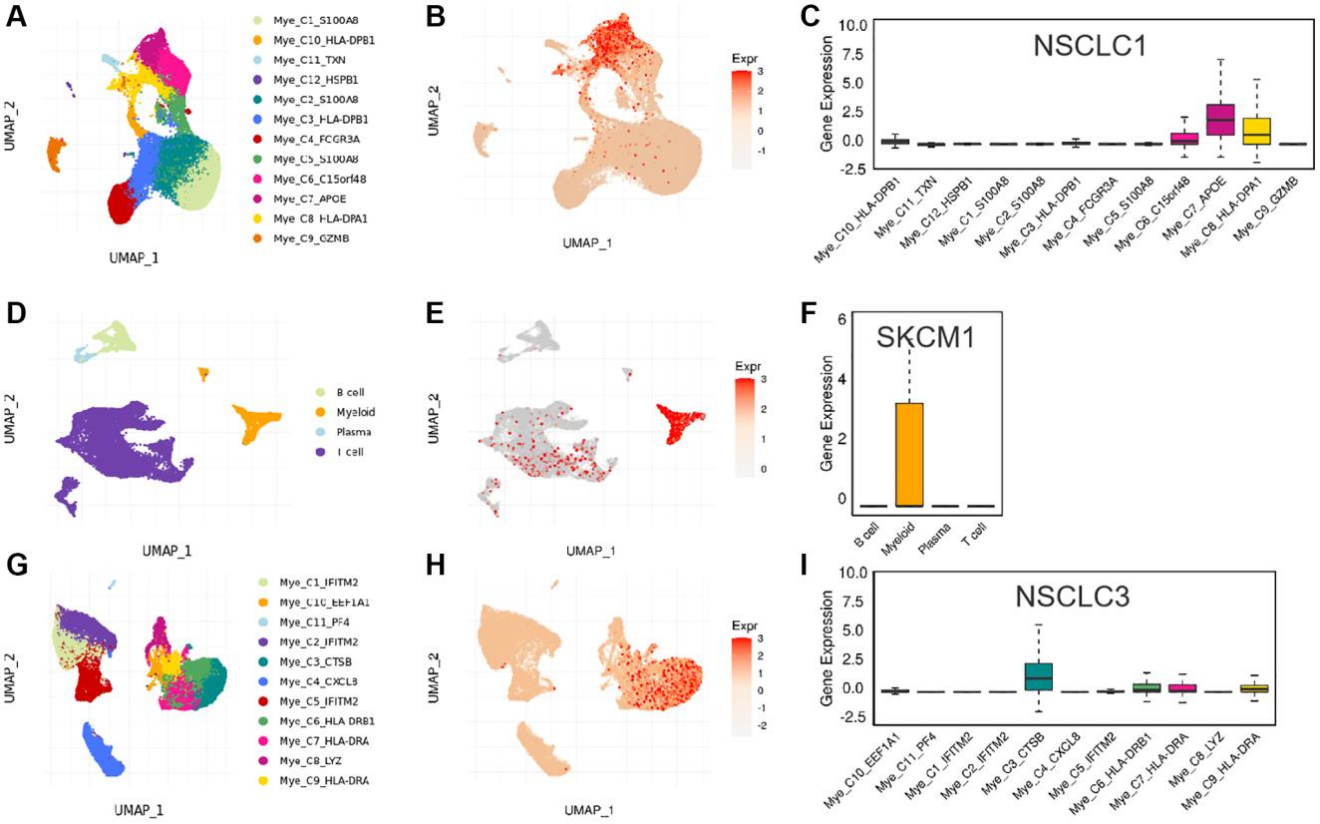
**The expression of SLAMF8 in various types immune cells.** (**A**–**C**) The distribution of various immune cells and the expression of SLAMF8 in different immune cells in NSCLC1 dataset. (**D**–**F**) The distribution of various immune cells and the expression of SLAMF8 in different immune cells in SKCM1 dataset. (**G**–**I**) The distribution of various immune cells and the expression of SLAMF8 in different immune cells in NSCLC3 dataset.

In addition, we used three data sets (HNSC_GSE103322, LIHC_GSE140228_Smartseq2 and NSCLC_GSE139555) downloaded in TISCH2 database to investigate the expression of SLAMF8 in TME-related cells. The highest expression of SLMAF8 was observed in monocytes/macrophages according to the results. Next, the analysis was conducted on the three data sets, revealing the quantities of different TME-associated cells and the presence of SLAMF8 across various cell types at a single-cell level (Figure 6). Furthermore, the IMMUcan SingleCell RNAseq Database was utilized to investigate the distribution of SLAMF8 expression in the tumor microenvironment (TME), as depicted in Figure 7. Results also displayed the expression of SLAMF8 in various cells of TME in NSCLC and LUAD, which were the top 2 most expression of SLAMF8 in TME-related cells. Lastly, TIGER database was applied to research the difference of SLAMF8 expression in pathway enrichment scores between different groups in each cell type. The top 3 types cancers were shown in Figure 8, and we found the SLAMF8 express in various types immune cells, such as Mye-C7_APOE, Myeloid and Mye-C3_CTSB.

### SLAMF8 expression is related to immune checkpoint genes and immune neoantigens

**Figure 9:**
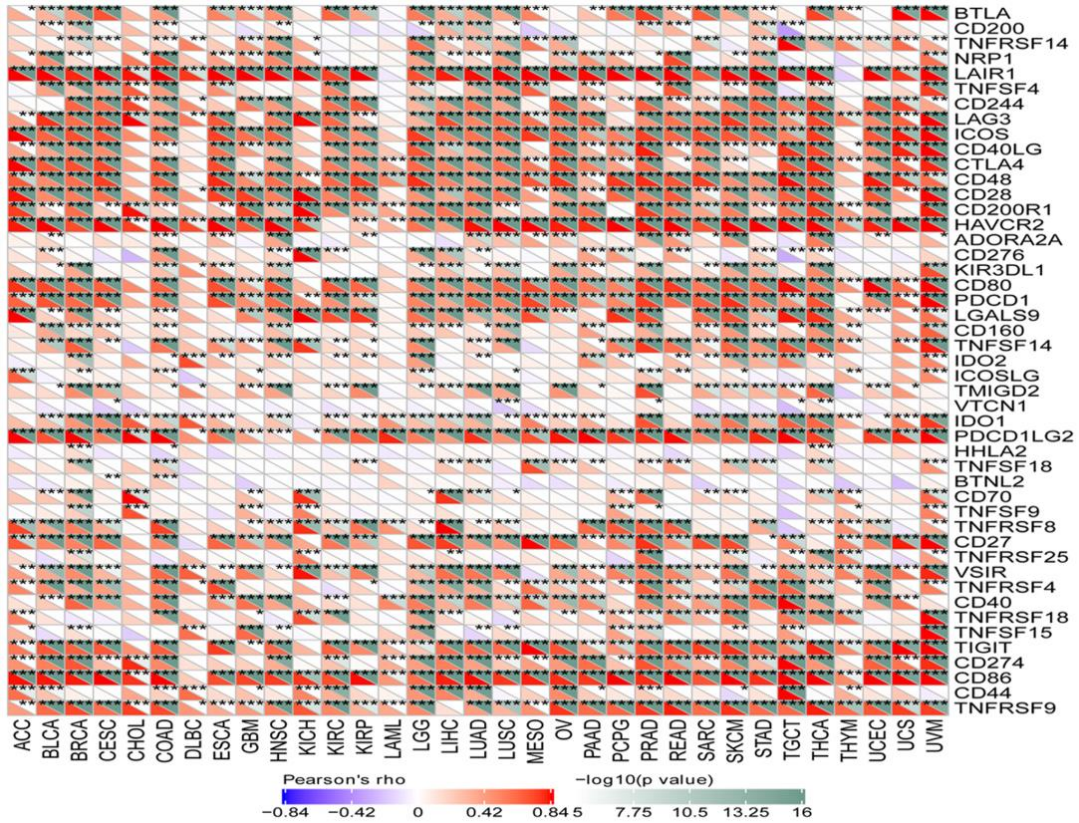
Correlation analysis between SLAMF8 expression and immune checkpoint genes in pan-cancer.

**Figure 10:**
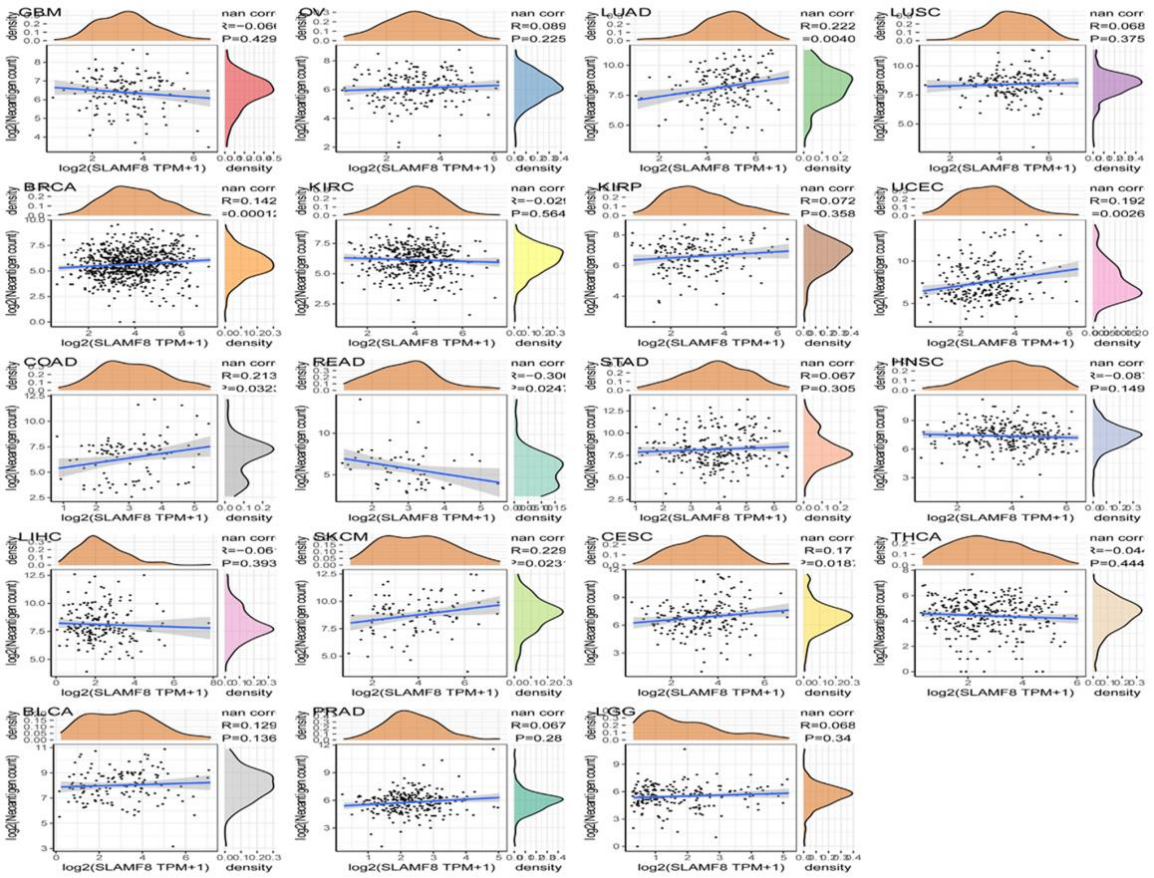
Correlation analysis between SLAMF8 expression and immune neoantigens in pan-cancer.

To investigate the correlation between SLAMF8 levels and the expression of checkpoint genes, information from over forty immune checkpoint genes, commonly observed in various cancer types, was gathered. The analysis results revealed a positive correlation between SLAMF8 expression and the levels of immune checkpoint genes in the majority of cancers, particularly in BRCA, LUAD, and UVM (Figure 9). The suggestion is that SLAMF8 might have a significant impact on controlling the expression of different genes related to immune checkpoints, thus influencing tumor immunity. Furthermore, the neoantigen quantities in each tumor type were assessed separately to determine the association between SLAMF8 levels and these neoantigens. According to Figure 10, the levels of SLAMF8 demonstrated a positive correlation with the quantity of neoantigens in LUAD (R = 0.222, *P* = 0.004), BRCA (R = 0.142, *P* < 0.001), UCEC (R = 0.192, *P* = 0.0026), COAD (R = 0.213, *P* = 0.0323), SKCM (R = 0.229, *P* = 0.0231), and CESC (R = 0.17, *P* = 0.0187). However, a negative correlation was observed in READ (R = 0.306, *P* = 0.0247).

### SLAMF8 is associated with tumor mutational burden as well as microsatellite instability

**Figure 11:**
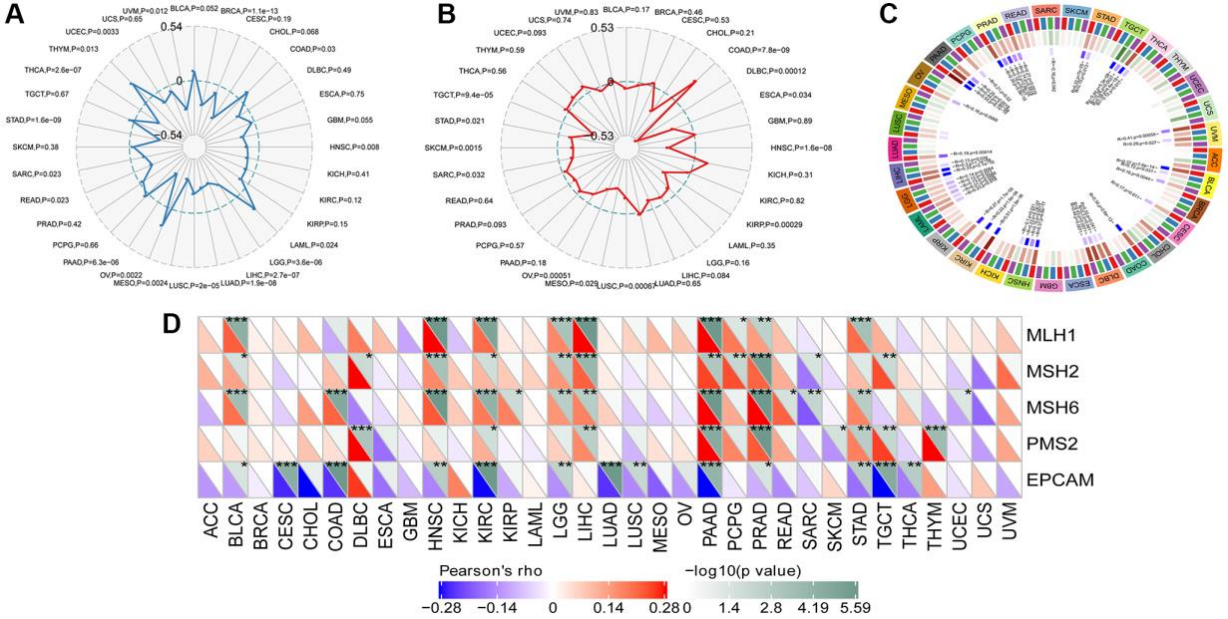
**Correlation analysis between SLAMF8 expression in pan-cancer and TMB, MSI, methyltransferases and MSI.** (**A**) Correlation analysis between SLAMF8 expression in pan-cancer and TMB. (**B**) Correlation analysis between SLAMF8 expression in pan-cancer and MSI. (**C**) Correlation analysis between the expression of SLAMF8 in pan-cancer and the expression levels of four types of methyltransferases. DNMT1 is colored red, DNMT2 is colored blue, DNMT3a is colored green, and DNMT3b is colored purple. (**D**) Correlation analysis between SLAMF8 expression in pan-cancer and DNA repair genes.

Tumor mutational burden (TMB) is considered a measurable indicator for quantifying the number of mutations in tumor cells. It represents the count of somatic mutations in the coding regions of a cancer cell genome, occurring on average at a rate of 1 million bases. Many types of mutations frequently involve the total count of nonsynonymous, which encompasses single nucleotide variations (SNVs) as well as small insertions/deletions. The relationship between SLAMF8 contents and TMB was explored in each tumor type by Spearman’s rank correlation analysis. Figure 11A demonstrated that SLAMF8 exhibited a positive correlation with TMB in COAD, LAML, OV, and THYM. Conversely, TMB showed an inverse association with SLAMF8 in BRCA, HNSC, LGG, LIHC, LUAD, LUCS, MESO, PAAD, READ, SARC, STAD, THCA, UCEC, and UVM.

Microsatellite instability (MSI) is the presence of a novel microsatellite allele in a tumor in contrast to neighboring healthy cells. This arises from alterations in the microsatellite’s length caused by the insertion or deletion of a repeating unit. Similarly, Spearman’s rank correlation was employed to evaluate the association between SLAMF8 and MSI. In COAD, SLAMF8 revealed a positive correlation with MSI, but in DLBC, ESCA, HNSC, KIRP, LUSC, MESO, OV, SARC, SKCM, STAD, and TGCT, it exhibited an inverse correlation (Figure 11B).

### SALMF8 is related to DNA mismatch repair genes as well as methyltransferase expression in pan-cancer

The findings depicted in Figure 11C indicate that nearly every MMRS gene exhibited a correlation with the levels of SLAMF8 in around half of the cancer types. This suggests that SLAMF8 might have a crucial function in the preservation of malignant cells by increasing the expression of genes related to DNA mismatch repair.

DNA methylation is the process of adding a methyl group to the 5′ carbon position of cytosine in genomic CpG dinucleotides, which is made possible by DNA methyltransferases. Analysis of the data revealed that SLAMF8 exhibited a positive correlation with the expression of four types of methyltransferases in over half of the cancer types (Figure 11D). The results demonstrated that SLAMF8 may play important role in modulating tumorigenesis along with progression through modulating epigenetic status in pan-cancer.

### SLAMF8 is related to mutation in pan-cancer

**Figure 12:**
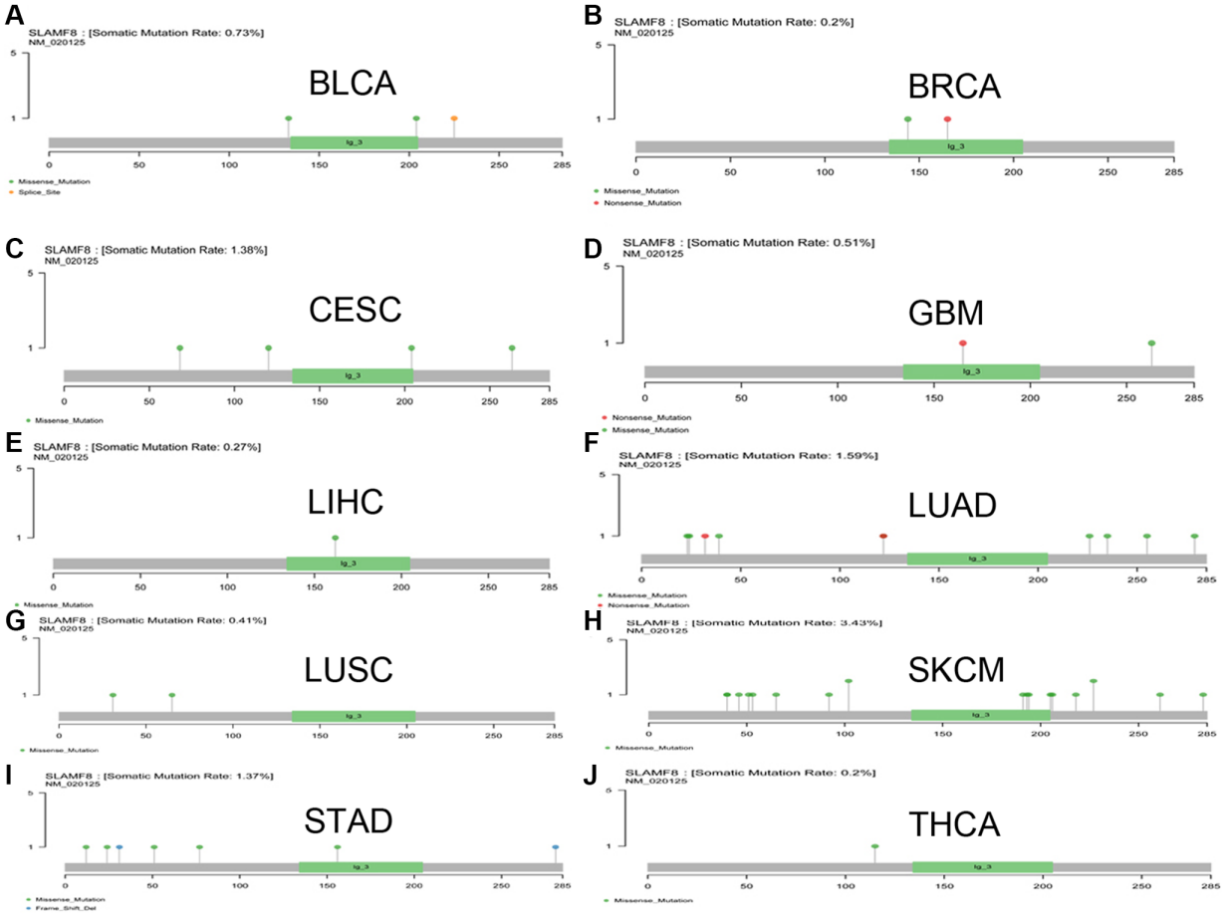
**The mutation of SLAMF8 gene in pan-cancer.** (**A**) The mutation of SLAMF8 gene in BLCA. (**B**) The mutation of SLAMF8 gene in BRCA. (**C**) The mutation of SLAMF8 gene in CESC. (**D**) The mutation of SLAMF8 gene in GBM. (**E**) The mutation of SLAMF8 gene in LIHC. (**F**) The mutation of SLAMF8 gene in LUAD. (**G**) The mutation of SLAMF8 gene in LUSC. (**H**) The mutation of SLAMF8 gene in SKCM. (**I**) The mutation of SLAMF8 gene in STAD. (**J**) The mutation of SLAMF8 gene in THCA.

The mutation data of pan-cancer downloaded from TCGA were collected, and the results illustrated the mutation site was most commonly located within ig-3 region. The somatic mutation rate in BLCA, BRCA, CESC, GBM, LIHC, LUAD, LUSC, SKCM, STAD, and THCA was 0.73%, 0.2%, 1.38%, 0.51%, 0.27%, 1.59%, 0.41%, 3.43%, 1.37%, and 0.2%, respectively, as shown in Figure 12.

### SLAMF8 is involved in the control of signaling pathways associated with cancer metabolism and tumor immunity

**Figure 13:**
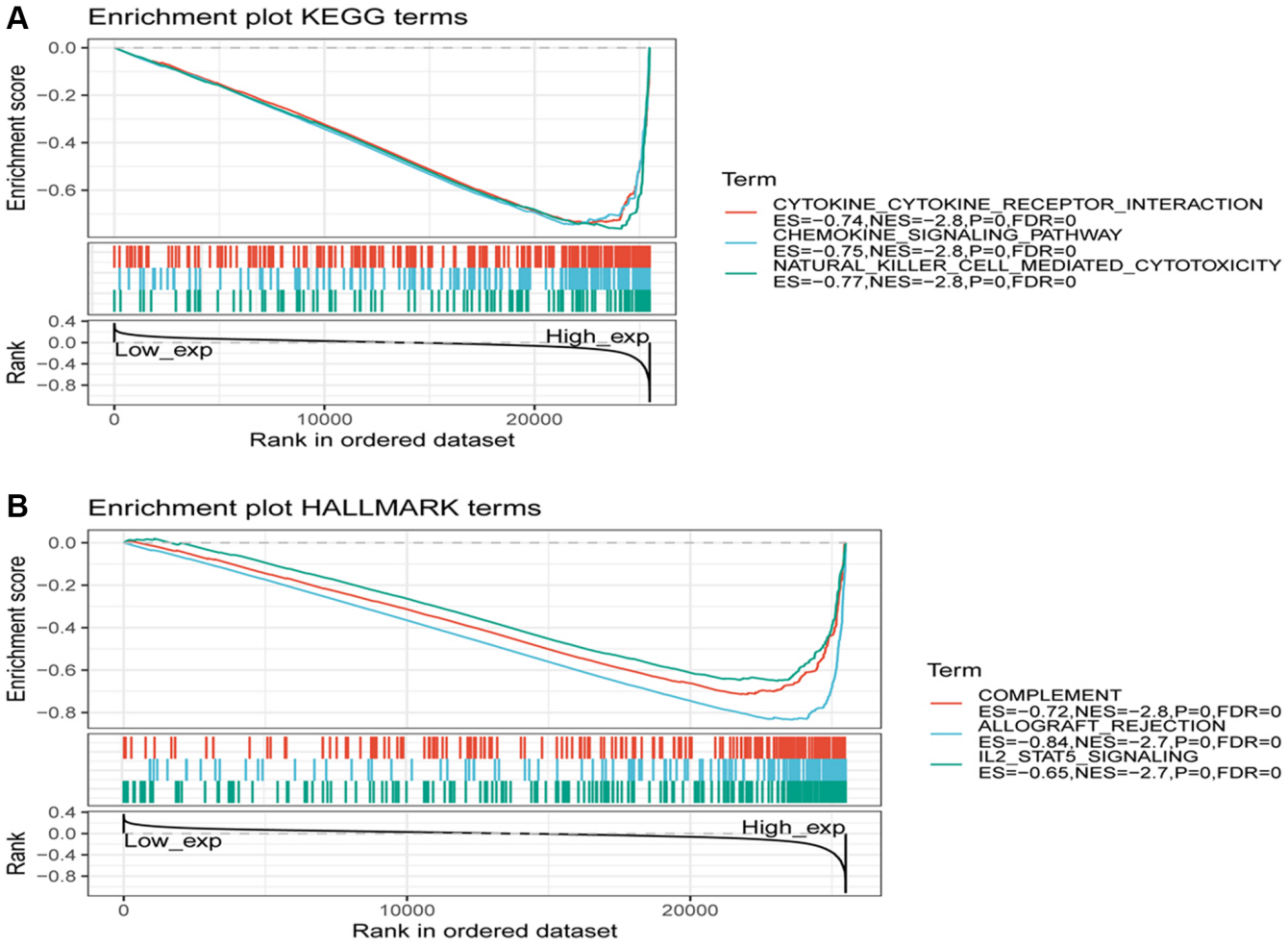
**Gene set enrichment analysis of SLAMF8 associated with signaling pathways in KEGG and hallmark datasets.** (**A**) Results of GSEA of SLAMF8 ranked in the top 3 for its association with signaling pathways in KEGG database. (**B**) Results of GSEA of the top 3 rankings of SLAMF8 correlation with signaling pathways in hallmark dataset.

To investigate the expression of the SLAMF8 gene in tumors, samples from human pan-cancer were categorized into two groups based on SLAMF8 content—high and low. To identify enriched signaling cascades within the two groups, Gene set enrichment analysis (GSEA) was utilized on the KEGG and hallmark data resources. The normalized enrichment score (Nes) permutation is used to present the top 20 most enriched signaling cascades or biological processes in Tables 1 and 2. Furthermore, Figure 13 depicts the top three signaling cascades that are highly enriched in both the high and low expression groups. Above results indicate that SLAMF8 has a vital function in regulating signaling pathways associated with tumor immunity and metabolism.

**Table 1:** The information of KEGG terms from top 20 GSEA enrichment analysis.

**Term**	**ES**	**NES**	**NP**	**FDR**	**FWER**
KEGG_CYTOKINE_CYTOKINE_RECEPTOR_INTERACTION	−0.74	−2.84	0	0	0
KEGG_CHEMOKINE_SIGNALING_PATHWAY	−0.75	−2.83	0	0	0
KEGG_NATURAL_KILLER_CELL_MEDIATED_CYTOTOXICITY	−0.77	−2.79	0	0	0
KEGG_T_CELL_RECEPTOR_SIGNALING_PATHWAY	−0.76	−2.63	0	0	0
KEGG_JAK_STAT_SIGNALING_PATHWAY	−0.68	−2.62	0	0	0
KEGG_CELL_ADHESION_MOLECULES_CAMS	−0.75	−2.60	0	0	0
KEGG_ANTIGEN_PROCESSING_AND_PRESENTATION	−0.80	−2.59	0	0	0
KEGG_TOLL_LIKE_RECEPTOR_SIGNALING_PATHWAY	−0.73	−2.59	0	0	0
KEGG_HEMATOPOIETIC_CELL_LINEAGE	−0.79	−2.56	0	0	0
KEGG_B_CELL_RECEPTOR_SIGNALING_PATHWAY	−0.75	−2.53	0	0	0
KEGG_NOD_LIKE_RECEPTOR_SIGNALING_PATHWAY	−0.75	−2.50	0	0	0
KEGG_AUTOIMMUNE_THYROID_DISEASE	−0.87	−2.49	0	0	0
KEGG_LEISHMANIA_INFECTION	−0.82	−2.45	0	0	0
KEGG_TYPE_I_DIABETES_MELLITUS	−0.89	−2.42	0	0	0
KEGG_PRION_DISEASES	−0.745	−2.37	0	0	0
KEGG_VIRAL_MYOCARDITIS	−0.76	−2.36	0	0	0
KEGG_FC_EPSILON_RI_SIGNALING_PATHWAY	−0.64	−2.34	0	0	0
KEGG_APOPTOSIS	−0.64	−2.33	0	0	0
KEGG_LEUKOCYTE_TRANSENDOTHELIAL_MIGRATION	−0.65	−2.33	0	0	0
KEGG_FC_GAMMA_R_MEDIATED_PHAGOCYTOSIS	−0.66	−2.32	0	0	0

**Table 2:** The information of HALLMARK terms from top 20 GSEA enrichment analysis.

**Term**	**ES**	**NES**	**NP**	**FDR**	**FWER**
HALLMARK_COMPLEMENT	−0.72	−2.76	0	0	0
HALLMARK_ALLOGRAFT_REJECTION	−0.84	−2.75	0	0	0
HALLMARK_IL2_STAT5_SIGNALING	−0.65	−2.69	0	0	0
HALLMARK_INFLAMMATORY_RESPONSE	−0.75	−2.65	0	0	0
HALLMARK_INTERFERON_GAMMA_RESPONSE	−0.84	−2.61	0	0	0
HALLMARK_KRAS_SIGNALING_UP	−0.64	−2.59	0	0	0
HALLMARK_IL6_JAK_STAT3_SIGNALING	−0.806	−2.589	0	0	0
HALLMARK_TNFA_SIGNALING_VIA_NFKB	−0.68	−2.37	0	0	0
HALLMARK_INTERFERON_ALPHA_RESPONSE	−0.84	−2.36	0	0	0
HALLMARK_APOPTOSIS	−0.58	−2.26	0	0	0
HALLMARK_PI3K_AKT_MTOR_SIGNALING	−0.59	−2.22	0	1.04E-04	0.001
HALLMARK_APICAL_SURFACE	−0.61	−2.15	0	8.10E-04	0.006
HALLMARK_APICAL_JUNCTION	−0.56	−2.10	0.002	0.002	0.011
HALLMARK_EPITHELIAL_MESENCHYMAL_TRANSITION	−0.63	−1.92	0.012	0.009	0.06
HALLMARK_COAGULATION	−0.49	−1.80	0.002	0.026	0.156
HALLMARK_MITOTIC_SPINDLE	−0.55	−1.80	0.008	0.024	0.156
HALLMARK_HYPOXIA	−0.43	−1.68	0.030	0.05	0.268
HALLMARK_G2M_CHECKPOINT	−0.58	−1.64	0.058	0.06	0.315
HALLMARK_ANGIOGENESIS	−0.51	−1.61	0.048	0.068	0.348
HALLMARK_TGF_BETA_SIGNALING	−0.49	−1.56	0.067	0.09	0.425

### Relationship between SLAMF8 expression and immune modulatory factors in pan-cancers

**Figure 14:**
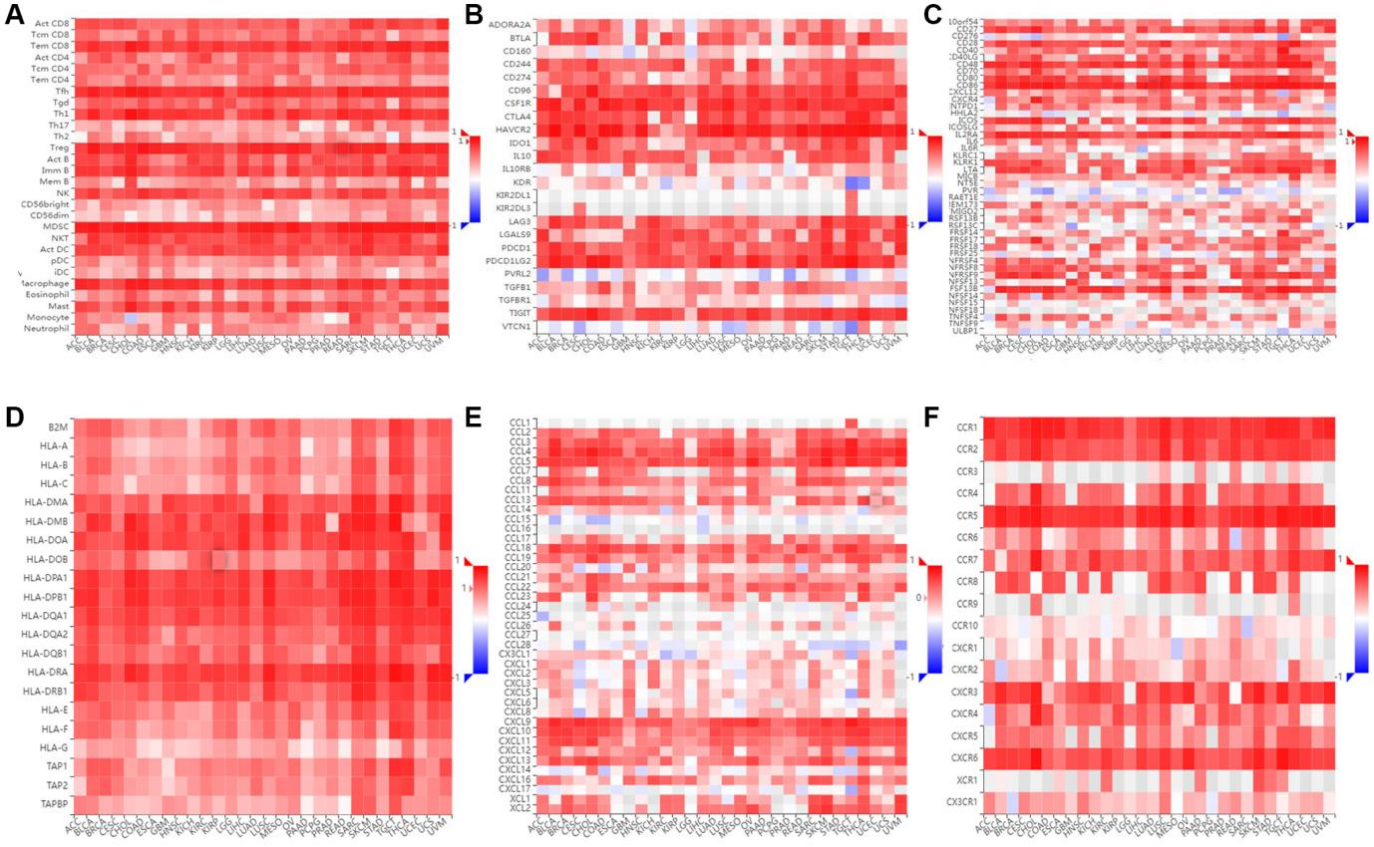
**Immunological correlation between SLAMF8 and immune modulatory factors in pan-cancers.** (**A**) Spearman correlation between expression of SLAMF8 and tumor-infiltrating lymphocytes, (**B**) Spearman correlation between expression of SLAMF8 and immunoinhibitors, (**C**) Spearman correlation between expression of SLAMF8 and immunostimulators, (**D**) Spearman correlation between expression of SLAMF8 and MHCs, (**E**) Spearman correlation between expression of SLAMF8 and chemokines, and (**F**) Spearman correlation between expression of SLAMF8 and receptors across human cancers.

The relationship between SLAMF8 and TILs was explored by using gene set variation analysis (GSVA) based on gene expression profile. Results as shown in Figure 14, SLAMF8 was significantly related to the various types of TILs (such as Tem CD8, Tfh, and MDSC) in the majority types of human cancers. Furthermore, by utilizing gene set variation analysis deponed on the gene expression profile, we discovered a correlation between the expression of SLAMF8 and Immunoinhibitors, Immunostimulators, MHCs, chemokines, and receptors across various types of cancer. According to Figure 14, the expression level of SLAMF8 was found to have a positive correlation with various Immunoinhibitors (like CSF1R, CD96, and HAVCR2), different types of Immunostimulators (such as CD27, IL2RA, and CD86), a wide range of MHCs (including HLA-DMA, HLA-DPB1, and HLA-DRB1), multiple chemokines (like CCL4, CCL18, and CXCL9), and numerous receptors (such as CCR1, CCR2, and CXCR6) in the majority of cancer cases. The aforementioned discoveries indicate that SLAMF8 might have a vital function in the regulation of tumor immunity across various types of cancer.

### SLAMF8 expression is related to immune and molecular subtypes in pan-cancers

**Figure 15:**
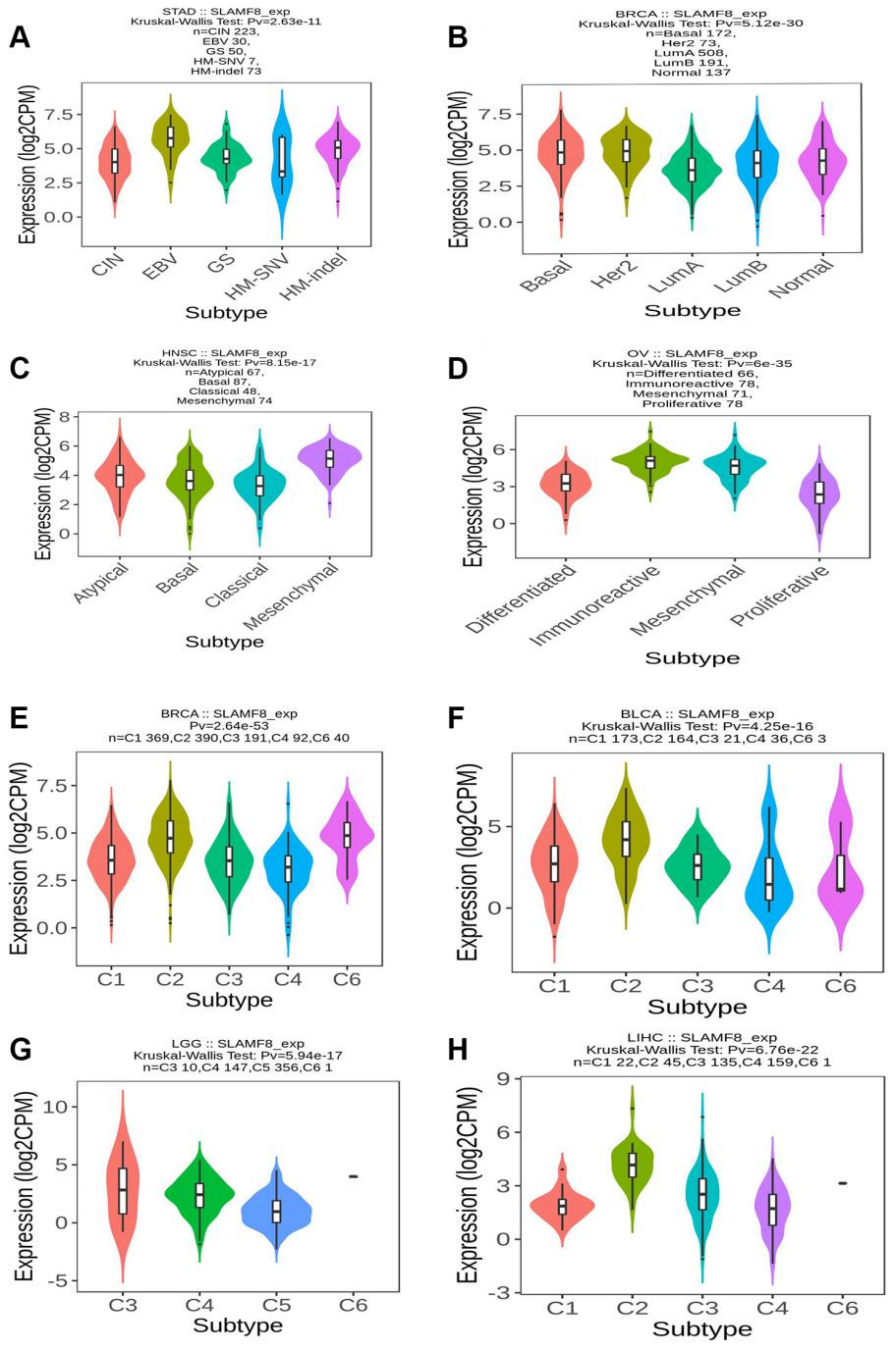
**SLAMF8 expression is related to immune and molecular subtypes in pan-cancers.** (**A**–**D**) The correlation between SLAMF8 expression and pan-cancer molecular subtypes. (**E**–**H**) The correlation between SLAMF8 expression and pan-cancer immune subtypes. Note: C1 (wound healing); C2 (IFN-gamma dominant); C3 (inflammatory); C4 (lymphocyte depleted); C5 (immunologically quiet); C6 (TGF-b dominant).

We utilized the TISIDB platform to investigate the influence of SLAMF8 expression on molecular subcategories and immune in pan-cancers. The results indicate that the expression of SLAMF8 differs across immune and molecular subtypes in different types of human cancers. Firstly, various molecular subcategories of malignancies displayed a remarkable association with SLAMF8 manifestation, and Figure 15A–15D presented the top four cancer types with the most noteworthy variances. Furthermore, the immune subtypes were categorized into six types: C1 (wound healing), C2 (IFN-gamma dominant), C3 (inflammatory), C4 (lymphocyte depleted), C5 (immunologically quiet), and C6 (TGF-b dominant). The data revealed that SLAMF8 expression was linked to different immune subtypes across various cancers. Additionally, SLAMF8 exhibited distinct expression patterns among immune subtypes within each cancer type, with the top four cancer types showing the most significant differences presented in Figure 15E–15H.

### Expression of SLAMF8 protein in cancer tissues

**Figure 16:**
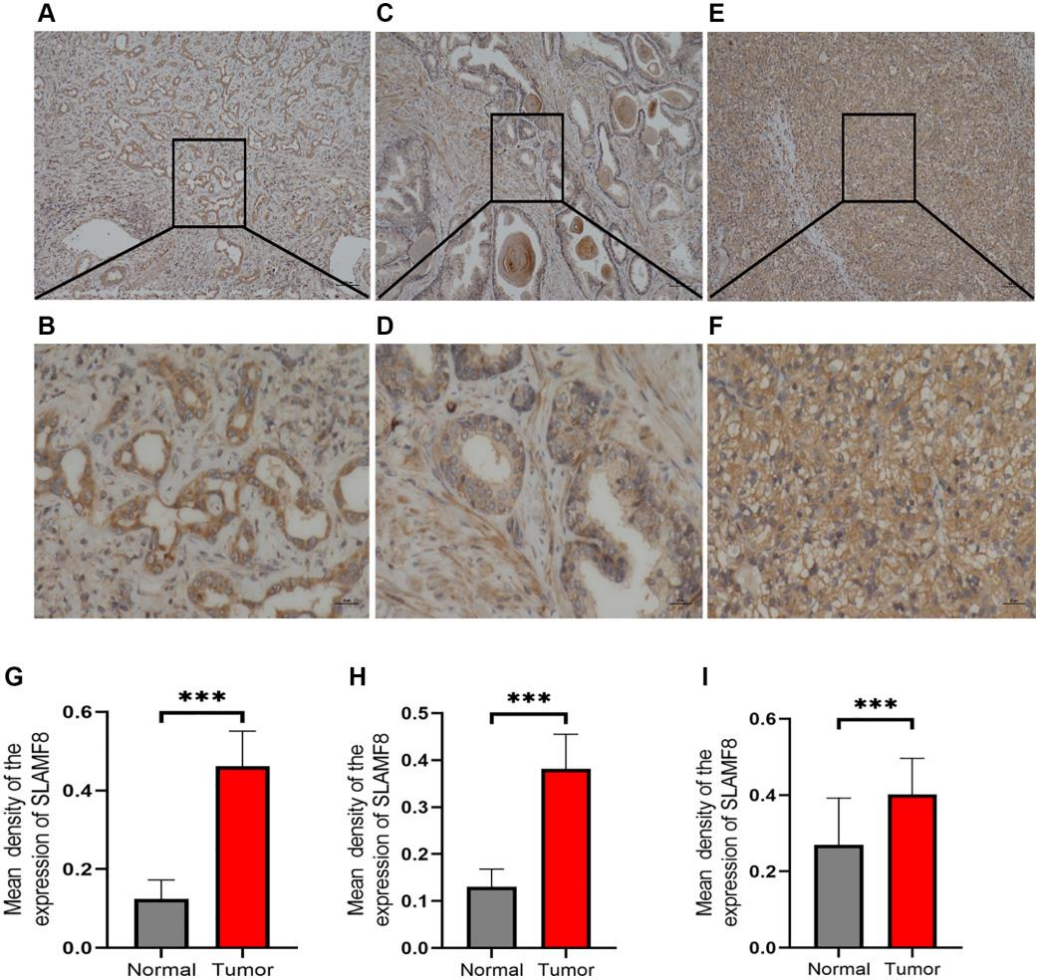
**Representative immunohistochemical staining of SLAMF8 in HCC, PRAD and KIRC tissues.** (**A**) Positive expression of SLAMF8 in HCC tissue, Magnification ×100. (**B**) Positive expression of SLAMF8 in HCC tissue, Magnification ×400. (**C**) Positive expression of SLAMF8 in PRAD tissue, Magnification ×100. (**D**) Positive expression of SLAMF8 in PRAD tissue, Magnification ×400. (**E**) Positive expression of SLAMF8 in KIRC tissue, Magnification ×100. (**F**) Positive expression of SLAMF8 in KIRC tissue, Magnification ×400. (**G**–**I**) Different expression of SLAMF8 in HCC, PRAD, KIRC tissue and matched adjacent noncancerous tissues. ^*^P < 0.05, ^**^P < 0.01, ^***^P < 0.001.

**Figure 17:**
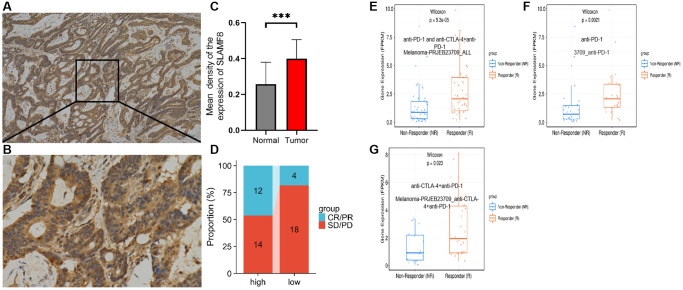
**Correlation analysis between SLAMF8 expression and immunotherapy.** (**A**) Representative immunohistochemical staining of SLAMF8 in STAD tissue, Magnification ×100. (**B**) Representative immunohistochemical staining of SLAMF8 in STAD tissue, Magnification ×400. (**C**) Different expression of SLAMF8 in STAD tissue and matched adjacent noncancerous tissues. ^***^P < 0.001. (**D**) Different therapeutic effectiveness of albumin paclitaxel combined with Sindillizumab between high expression of SLAMF8 and low expression of SLAMF8 in 48 patients who were recurrent after radical gastric cancer surgery. (**E**–**G**) Correlation analysis between SLAMF8 expression and the therapeutic effectiveness of immunotherapy based on Melanoma-PRJEB23709_ALL, 3709_ANTI-PD-1 and Melanoma-PRJEB23709_anti-CTLA-4+anti-PD-1 datasets downloaded from TIGER database.

To comprehensively determine the SLAMF8 expression in cancers, as sample, immunohistochemistry was applied to explore the expression of SLAMF8 in HCC (30 cases), PRAD (30 cases), and KIRC (30 cases) tissues and corresponding surrounding tissues. The findings indicated that SLAMF8 exhibited a significant increase in cancer tissues (Figure 16), suggesting its crucial involvement in cancer development. Furthermore, we use the TIGER database to analyze the relationship between SLAMF8 expression and tumor immunotherapy effectiveness (Figure 17), and results demonstrated high SLAMF8 expression was markedly associated with the therapy effectiveness for cancers. Moreover, we found the treatment effectiveness of albumin paclitaxel combined with Sintilimab (anti-pd1) is significantly increased among the patients with STAD with high expression of SLAMF8 and postoperative recurrence (48 cases) which verified the results of the database. Therefore, our findings suggest that SLAMF8 may have a crucial role in the context of tumor immunotherapy.

## DISCUSSION

SLAMFs, which are found on a variety of hematopoietic cells including macrophages, T cells, and NK cells, play a crucial role in controlling the actions of immune cells [17]. SLAMF8, the eighth gene in the SLAM family gene series, plays a role in encoding a set of cell surface proteins known as the CD2 protein family, which is involved in the activation of lymphocytes [18, 19]. Furthermore, SLAMF8 plays a vital function in the B cell lineage and controls signaling via the B cell receptors [20]. Previous studies have shown that SLAMF8 was correlated with immune-related diseases [5, 6, 8, 21], however, few studies explored the function and efficacy of SLAMF8 in pan-cancers.

In this investigation, our initial observation revealed elevated expression of SLAMF8 in pan-cancers, and heightened SLAMF8 expression is linked to a poorer prognosis in the majority of cancer types. Furthermore, the IHC experimental result shown SLAMF8 expressed higher than adjacent tissue in HCC, PRAD and KIRC. Besides, the data herein demonstrated the mutation of SLAMF8 gene in various types of cancers. These findings suggest that SLAMF8 might have a pivotal role in the processes of tumor tumorigenesis and progression in pan-cancers.

The tumor microenvironment (TME) includes the surroundings of a tumor mass, which consist of not just cancer cells but also stromal cells, new blood vessels, immune cells, and extracellular matrix. Recent studies have proved that TME was closely associated with tumor progression [22, 23]. Tumor infiltrating lymphocytes are lymphocytes around tumor cells in TME, which regarded as an independent predictor of evaluating cancers prognosis and immunotherapeutic efficacy [24, 25]. According to our findings, we noticed a favorable connection between the manifestation of SLAMF8 and different categories of tumor-invading lymphocytes, such as stimulated CD8, CD8 with memory, and Treg cells (regulatory T cells). The single-cell analysis based on TISCH2, IMMUNcan and TIGER database shown SLAMF8 expression widely distributed in immune-related cells. It implied that SLAMF8 may be regarded as a potential target gene in the tumor immune-related mechanisms researches. For example, our studies shown that SLAMF8 is positively associated with myeloid-derived suppressor cells (MDSC) in almost all types of cancer, which have been proved be a vital role in mediating immunosuppression in TME. In our study, we have gathered a total of 47 different checkpoint genes. We observed a strong correlation between SLAMF expression and various important checkpoint genes in most cancer types, including CD274 (PD-L1) and LAG3. When the activity of the T cell receptor reaches a specific threshold, PD-L1 interacts with its corresponding receptor PD-1 to inhibit T cell proliferation and the secretion of cytokines. The TME is affected by this process, which aids in preserving peripheral tolerance and hindering the immune activity of T cells [26, 27]. LAG3 acts as an inhibitory factor for T cells, and past studies have demonstrated that inhibiting LAG3 can enhance the proliferation and functional capabilities of cytotoxic T lymphocytes. LAG3 is expressed at the same time as PD-1 in infiltrating lymphocytes within tumors, and the combination of LAG3 and PD-1 blockade has the possibility to improve tumor control or trigger regression [28, 29]. Our research revealed that SLAMF8, PD-1L, and LAG3 exhibited a certain pattern of expression, indicating their potential role in modulating tumor aggressiveness via a common pathway. Nevertheless, further investigations are required to delve into the underlying mechanisms. TMB and MSI are focal points of research in the cancer field, significantly influencing both tumor characteristics and the survival outcomes of patients [30, 31]. In current study, we found SLAMF8 expression was negatively correlated to MSI and TMB in the majority of cancers. Hence, we speculated that aberrant SLAMF8 expression may perform repressive effect in the regulation of neo-antigen, more researches are needed to launch to explore the Specific mechanism. Tumor immunotherapy is a kind of effective treatment for cancers, which recovers the antitumor immunoreaction via rebooting and maintaining the tumor-immune circulation. During our recent inquiry, we discovered a significant correlation between SLAMF8 and specific genes that have been confirmed as target genes in cancer immunotherapy for different forms of cancer. These genes encompass immunoinhibitors like CD96 [32, 33], immunostimulators such as CD27 [34], major histocompatibility complexes like HLA-DRA [35, 36], chemokines like CCL5 [37, 38], and receptors including CCR1 [39, 40]. Furthermore, according to our findings, we observed a strong association between elevated SLAMF8 levels and the efficacy of albumin-bound paclitaxel in combination with Sindillizumab in STAD patients who experienced recurrence following radical resection. As a result, we propose that SLAMF8 has the potential to be a valuable contender in the progress of tumor immunotherapy.

To explore the functional role of SLAMF8 in pan-cancer, including both groups with high and low SLAMF8 expression, gene set enrichment analysis was utilized. The results demonstrated the participation of SLAMF8 in multiple signaling pathways, such as the signaling pathway of cytokine-cytokine receptor interaction, chemokine-signaling pathway, and complement signaling pathway. The high-expression subgroup of SLAMF8 exhibited the highest enrichment scores in these signaling cascades, indicating a positive modulation by elevated SLAMF8 expression. Based on these discoveries, we can deduce that SLAMF8 might have a pivotal function in boosting immune response, impacting the survival of tumor cells, and stimulating their growth through the upregulation of related signaling pathways.

## CONCLUSION

In conclusion, SLAMF8 exhibits up-regulation in tissues across various types of cancer. Elevated SLAMF8 expression is notably associated with an unfavorable clinical prognosis in some cancers. Factors like TMB, MSI, MMRs, and DNA methylation could potentially impact the dysregulation of SLAMF8 expression in various cancers. Additionally, the significant correlation between SLAMF8 and the tumor immune microenvironment highlights its potential as a valuable target gene for tumor immunotherapy.
